# Comparative Analysis of Major Machine-Learning-Based Path Loss Models for Enclosed Indoor Channels

**DOI:** 10.3390/s22134967

**Published:** 2022-06-30

**Authors:** Mohamed K. Elmezughi, Omran Salih, Thomas J. Afullo, Kevin J. Duffy

**Affiliations:** 1 The Discipline of Electrical, Electronic and Computer Engineering, University of KwaZulu-Natal, Durban 4041, South Africa; afullot@ukzn.ac.za; 2 Institute of Systems Science, Durban University of Technology, Durban 4000, South Africa; kevind@dut.ac.za

**Keywords:** wireless communications, channel modeling, path loss, propagation characteristics, machine learning, neural network, random forest, regression, 5G, 6G

## Abstract

Unlimited access to information and data sharing wherever and at any time for anyone and anything is a fundamental component of fifth-generation (5G) wireless communication and beyond. Therefore, it has become inevitable to exploit the super-high frequency (SHF) and millimeter-wave (mmWave) frequency bands for future wireless networks due to their attractive ability to provide extremely high data rates because of the availability of vast amounts of bandwidth. However, due to the characteristics and sensitivity of wireless signals to the propagation effects in these frequency bands, more accurate path loss prediction models are vital for the planning, evaluating, and optimizing future wireless communication networks. This paper presents and evaluates the performance of several well-known machine learning methods, including multiple linear regression (MLR), polynomial regression (PR), support vector regression (SVR), as well as the methods using decision trees (DT), random forests (RF), K-nearest neighbors (KNN), artificial neural networks (ANN), and artificial recurrent neural networks (RNN). RNNs are mainly based on long short-term memory (LSTM). The models are compared based on measurement data to provide the best fitting machine-learning-based path loss prediction models. The main results obtained from this study show that the best root-mean-square error (RMSE) performance is given by the ANN and RNN-LSTM methods, while the worst is for the MLR method. All the RMSE values for the given learning techniques are in the range of 0.0216 to 2.9008 dB. Furthermore, this work shows that the models (except for the MLR model) perform excellently in fitting actual measurement data for wireless communications in enclosed indoor environments since they provide R-squared and correlation values higher than 0.91 and 0.96, respectively. The paper shows that these learning methods could be used as accurate and stable models for predicting path loss in the mmWave frequency regime.

## 1. Introduction

Mobile communication has proliferated since its debut due to its flexibility and convenience. Due to the continuous evolution of communication technologies and the exponentially increasing demand for higher mobile data traffic, research has been focused on the frequency regime above 6 GHz to overcome the congestion of the previous bands (below 6 GHz) and to cope with the requirements of the fifth-generation (5G) wireless system and other high-speed multimedia services [1,2,3,4,5].

Large-scale fading models play a vital role in optimizing base station deployments, estimating radio coverage, and characterizing the radio environment to quantify the performance of wireless communication systems [6]. Furthermore, efficient and reliable determination of crucial factors, such as the signal field strength, carrier-to-interference (C/I) ratio, and signal-to-noise ratio (SNR), can be achieved if in-depth knowledge of propagation loss is provided [7]. As a result, for network planning and implementation of wireless communication systems, radio propagation channel characteristics in various environments are required [8].

The performance of wireless communication systems is influenced by radio propagation in physical environments since the radio waves often experience fading. The wirelessly propagated signals from any communication system’s transmitting antenna(s) suffer from attenuations over distances and frequencies, which are well-known as large-scale fading and small-scale fading. In addition, the signals also experience losses because of atmospheric conditions and surrounding physical objects, leading to multipath propagation since the receiving antenna(s) receives the signals mainly from reflections, diffractions, and scattering mechanisms [9,10]. These multipath effects result in signal power fluctuation and increase the uncertainty of received signal power [11]. This work mainly focuses on developing large-scale path loss models that are crucial for estimating radio coverage, allocating frequencies properly, optimizing base stations, and identifying the most suitable antennas [6,12].

Radio propagation models representing path loss are essential to ensure high-quality services and accurate signal coverage predictions in mobile communication networks. Accordingly, researchers have accelerated their efforts to provide reliable models for various environments and scenarios over a wide range of frequency regimes to assist network engineers in designing reliable future wireless networks and accurate link budget calculations. Moreover, accurate predictions could be beneficial in radio resource management schemes that aim to meet specific Quality-of-Service (QoS) criteria [13,14,15,16,17,18,19,20].

Fundamentally, traditional approaches to path loss prediction modeling are deterministic, empirical, and stochastic. Deterministic path loss prediction models are site-specific and require sufficient information of the propagation’s environments. These models are often related to 3-D map propagations, such as the ray-tracing models. Moreover, these deterministic models repeat their calculations when the environment changes; therefore, they have high computational complexity. The empirical path loss models are based on measurements and observations, such as the Hata model and the COST 231 model. These models are easier to apply; however, they are time-consuming since they require massive measurement campaigns in specific environments and communication scenarios. Further, in terms of prediction accuracy, these models provide less accuracy than deterministic models [6,12,21]. Many other well-known empirical path loss prediction models are derived based on the criteria of the minimum mean square error (MMSE) between the models and the measurement data to provide the best fit of these models as a function of the separation distance between the transmitting and receiving antennas in the logarithmic scale. They include the single-frequency close-in (CI) free space reference distance model and the single-frequency floating-intercept (FI) model [22,23,24,25,26,27,28,29,30]. Stochastic path loss models have probability distributions and assumptions to be considered in the models’ equations. These models suffer from limited precision because of some mathematical expressions since the communications environments are considered random variables [6].

Multi-frequency path loss prediction models are receiving more attention from some researchers in recent times to develop accurate and stable path loss models for future wireless systems over a wide range of frequency regimes [6,31]. However, these models face the same problems mentioned above as the single-frequency models. There are two main problems in using the previous empirical models: the first problem is that these models are accomplished by a large amount of measurement in a certain environment to obtain a specific model that works for a particular environment at a specific frequency band, which is obviously time-consuming. The second problem is the limited prediction accuracy (the models do not fit the measurement data with a deficient prediction error) provided by these log-distance path loss models in some specific regions. Moreover, the use of the traditional linear models for predicting path loss is not sufficient to capture the path loss behavior accurately in higher frequency bands that are adopted to cope with the emerging demands of new wireless technologies. Accordingly, innovative methods that provide reliable modeling and prediction of the wireless propagation channels are highly needed, especially for complex environments of radio wave propagation that have a severe influence on the quality of wireless communication systems.

Machine learning (ML) is a set of approaches for making predictions based on datasets and modeling algorithms. ML-based methods are now used in various disciplines, including speech recognition, image identification, natural language processing, and computer vision. In many telecommunication fields, the research based on ML of various topics such as propagation loss prediction, channel decoding, signal detection, and channel estimation has already made significant progress [32,33,34]. All ML methods rely on the type of information (input features) that is used for the training. ML methods can be classified as supervised learning and unsupervised learning. For classification or regression issues, supervised learning is used to learn a function or relationship between inputs and outputs. Unsupervised learning, on the other hand, is the process of extracting hidden rules or connections from unlabeled data. Path loss prediction can be viewed as a supervised regression problem that ML methods can handle [11,35,36,37,38]. Path loss prediction models based on machine learning algorithms are promising to overcome the time consumption in traditional linear path loss models that depend mainly on measurement campaigns at new frequency bands in specific outdoor and indoor environments and communication scenarios and/or simulation-based methods, such as ray-tracing techniques [6]. ML-based algorithms have been successfully used to assist to predict the path loss in several operating environments [6,7,11,13,21,36,37,38,39,40,41,42,43,44,45,46,47,48,49,50,51,52,53,54,55,56]. Furthermore, unlike traditional models, ML-based path loss prediction models can provide reliable generalizations on the propagation environment [42].

There are many distinct types of machine learning algorithms, each with its own structure. Our objective is to see if these models can offer reliable prediction results at mmWave frequencies for a specific environment, e.g., a typical indoor corridor that can be viewed as an air-filled rectangular waveguide with huge dimensions compared to the wireless signals’ wavelength [57]. To the best of our knowledge, this is the first effort in predicting the path loss at frequency bands higher than 6 GHz for typical indoor corridor environments based on several ML methods. Path loss prediction models with the highest possible accuracy are vital for such environments since the trend is to rely on indoor channels for future wireless networks. Therefore, the primary motivation of this work is to evaluate the feasibility and prediction accuracy of various machine-learning-based models for predicting path loss in indoor corridor environments. Eight machine learning methods are presented and evaluated in this work. The methods include multiple linear regression (MLR), polynomial regression (PR), decision tree (DT), random forest (RF), support vector regression (SVR), K-nearest neighbor (KNN), artificial neural network (ANN), and long short-term memory (LSTM) artificial recurrent neural network (RNN), RNN-LSTM. The study is based on actual measurement data collected in a typical indoor corridor environment at three frequency bands from the SHF band, namely 14, 18, and 22 GHz. Our previously published work sufficiently describes the measurement campaigns and the channel sounder setup [10].

The rest of this paper is structured as follows. First, a review of the existing related works aimed at predicting the path loss based on ML algorithms is presented in Section 2. Then, Section 3 discusses the preparation of the raw data, hyperparameters tuning, justification of the models’ stability and the evaluation metrics. Section 4 describes the methods adopted for this study. The main results of this research work are discussed in Section 5. Finally, Section 6 concludes the research findings of this paper.

## 2. Related Works

Many recent research studies have adopted the methodology of using neural networks for the prediction of path loss based on measurement data in a specific frequency band for a specific environment and communication scenario; they then compare their prediction models with the traditional models in terms of accuracy and prediction error analysis. Various supervised learning approaches, such as the ANN [48,49,58,59], support vector machine (SVM) [37,60], KNN [38], and RF [38], have been successfully used to construct path loss models. Recently, it was reported that deep learning methods such as the deep neural network (DNN) and ANN provide better prediction results compared to the traditional path loss models [8,12]. Moreover, in [36], ANN-based path loss prediction models provided better performance than ML-based models, including the RF and SVR models. Further, the ANN-based model was proven to be superior to the log-distance model in the same study. In addition, the authors of [48,49,59] offered prediction models using ANN and showed more accuracy than other empirical models in terms of path loss prediction. A vision for developing real-time prediction models for path loss can be found in [59]. In [6], a DNN multi-frequency path loss model was analyzed and compared with the alpha-beta-gamma (ABG) path loss model. The results show that the DNN-based model is far better than the ABG model based on the results of prediction error analysis metrics.

Generally, any neural network is made up of nodes, which are processing components that are tightly linked and organized in layers. They have the capacity to describe any function that is given to them from the raw datasets. Consequently, setting up closed-form equations to map the input features into output target(s) is unnecessary for neural networks, unlike traditional methods. It is essential to use appropriate features as inputs to train and test ML-based models since they make the model more efficient and adaptable while reducing the solution complexity [11]. The input features of the ANN-based path loss model for unmanned aerial vehicles (UAVs) at 1800 and 2100 MHz were distance, clutter height, altitude, longitude, latitude, and elevation, as reported in [21]. In another work, the features adopted for ML-based path loss prediction in an urban environment included the Tx–Rx separation distance, as well as building information such as height, thickness, and distance away from the antenna [37]. In [61], the input for a successful deep convolutional neural network was 2D satellite images to provide reliable LTE signal quality metrics calculations. In [62], an ANN model with 48 neurons in a single hidden layer and a hyperbolic tangent activation function (also called Tanh activation function) has provided the best performance over several empirical path loss models such as COST-231, HATA, ECC-33, and EIGI. The input features adopted for this single-layered feed-forward neural network were distance, clutter height, altitude, elevation, latitude, and longitude, while the network had one output layer, which represents the path loss. Another work adopted an ANN model with two hidden layers to predict the path loss at the 1800 MHz frequency band for smart campus environments [45]. The main result obtained from that work is that the ANN model outperforms the RF-based model for such environments. Similar results provided the ANN model’s quality over the RF model since it can extract relevant input features of communication environments [54].

The author of [40] proposed a multilayer perceptron (MLP) feed-forward neural network model for predicting path loss. The model was based on 11 input features and 2 hidden layers with the use of the Tan-sigmoidal as an activation function. The 11 features selected were the Tx–Rx separation distance, operating frequency, transmitter terrain height, receiver terrain height, transmitting antenna height, average clutter height, %water, %building, %plain, %road, and %trees. At the same time, the path loss was the only output of this ANN model. The results obtained from this research reveal high prediction accuracy in predicting path loss since the degree of correlation values were higher than 0.94 for the model designed. However, the model’s complexity is high since it needs comprehensive knowledge to train from many features, limiting the adoption of such models. A comparative analysis of conventional, ML, and DNN-based methods for path loss prediction proved that the latter has the best performance since higher prediction accuracy was achieved than the other methods [48]. Using many layers in deep learning provides extraction of the features from high-dimensional datasets via training; this benefit is often not possible in using traditional models [63]. Moreover, DNN-based models do not rely on a predefined mathematical formula to represent the model as the conventional models do [64]. These DNN-based methods have been applied to many communication environments, such as rural, urban, and suburban areas [6]. In [65], DNN-based models were developed for five environments under the category of urban, dense urban, suburban, dense suburban, and rural for a specific frequency regime and proved its prediction accuracy.

In contrast, in [66], DNN-based models were proposed over a wide range of frequencies from the ultra-high frequency (UHF) band to the SHF band. The previous efforts’ datasets for the line-of-sight (LOS) and non-line-of-sight (NLOS) communication scenarios were based on measurement campaigns. Other studies considered the data from the satellite images, such as in [8,12,35]. The Tx–Rx separation distance and the operating frequency have been selected as the only two features for ANN-based path loss prediction models for an urban area at 3.4, 5.3, and 6.4 GHz and for a suburban environment at 450, 1450, and 2300 MHz [21]. The proposed ANN models achieved higher prediction accuracy than the other well-known models, such as the CI model, the Gaussian process model, and the two-rays model. Aside from the frequency and distance parameters, wall and floor attenuation are also utilized as input features for the ANN model to predict path loss in a multi-wall environment [26]. However, considering multi-dimensional regression to predict the path loss based on several highly correlated input features such as distance, frequency, antenna height, and other factors is still lacking in the literature. Because the associated inputs are uncertain, several candidate functions are used in the regression. Many of these characteristics, on the other hand, would be superfluous or unnecessary. Furthermore, most input features lack the capacity to discriminate for prediction. Using dimensionality reduction techniques, such as principal component analysis (PCA) or singular value decomposition (SVD), the input data could be transformed into a smaller representation set of features in order to create a decent estimate [21]. Dimensionality reduction is used to minimize the number of features in an input dataset while preserving as much helpful information as feasible.

For the ANN, some studies suggest that a neural network is a deep network with only one hidden layer. This theory supports the path loss prediction problems proposed by these studies [12,44,64]. Other efforts stated that having two or three hidden layers for the ANN model is enough to provide an accurate approximation of the non-linear relationship between the input features and the output target of the model [21]. Note that the complexity of the models can be reduced by adopting a small number of hidden layers taking into account the tradeoff between the models’ accuracy and complexity. The diversity of these studies in the open literature justifies no specific rule to provide the optimum size (for example, the number of hidden layers and the number of neurons per hidden layer) of the ML-based models. Of course, all of that depends on the training dataset given from measurement campaigns and/or simulation tools, and this dataset depends on the specific environments and scenarios of communication. As a result, the model’s hyperparameters, such as learning rate, activation function, and optimizers, are experimentally selected to provide the best model’s performance. Some studies introduced hyperparameter-tuning techniques, such as grid search and random search to overcome the time consumption of manually selecting the optimum models’ parameters [36].

The authors in [11] developed an RF-based path loss model benefiting from the fact that this method is based on a massive number of regression trees and that the path loss is also a regression problem, as mentioned earlier. The data used to train the model comes from four typical mobile communication terrains. The outcome of this study proved that the proposed RF model performed better than traditional wireless propagation models as well as a DNN model after constructing relevant features. The average root-mean-square error (RMSE) for the RF-based model was 6.106 dB for all types of terrains selected. A combined model for path loss and shadowing based on the ANN multilayer perceptron (ANN-MLP) was developed in [21]. The shadowing impact was analyzed based on the Gaussian process to provide the variance (or standard deviation) from the training dataset. This technique will help calculate the shadowing attributed to the shielding effect of buildings, mountains, and other objects that exist in the communication channel between the Tx and Rx. The results provided show the usefulness of the model in predicting the propagation loss.

A path loss prediction model was proposed for urban environments using the SVM method [47]. The input features selected were the Tx–Rx separation distance, vertical and horizontal antenna attenuations as system-specific parameters, as well as latitude, longitude, and terrain elevation as environment-specific parameters made up of six features. A similar work documented in [67] used the same environment-specific parameters in a deep-learning-based model. Both [47,67] show that the proposed ML-based models provide higher efficiency than other analytical models.

Two forms of probabilistic path loss predictors for a specific communication environment are reported and investigated in [41]. The first approach utilizes Bayesian learning to get the posterior distribution of an analytical model’s parameters and produces a path loss value prediction. A probabilistic neural network is used in the second technique to obtain the parameters of analytical distributions. The authors also studied the effect of changing the amount of data available for training on the ML proposed models’ performance. The prediction capacity for the models was measured in terms of the total variation distance (TVD) and Kullback–Leibler (KL). It is to be noted from their results that the mixture density neural network (MDN) model has more accuracy in describing the path loss than the Bayesian learning model. However, the latter provided better data efficiency than the MDN model. These probabilistic path loss models are beneficial since they overcome the problem of classifying the propagation path between the transmitting and receiving antennas as LOS or NLOS, given the probability of having a clear LOS connection between the Tx and Rx is already considered in the model. Hence, the knowledge of the LOS and NLOS communication scenarios is not required for such models. It is worth noting that the MDN model is basically a combination of a conventional Neural Network (NN) and a mixture model. This model has the ability to provide a distribution of the path loss values instead of point estimates. The neural network adopted for this work has only one input feature; that is, the separation distance between the Tx and Rx, one hidden layer of 64 neurons with the use of the rectified linear unit (ReLU) as an activation function, and an output layer that represents the path loss. It is clear from the previously documented works that there is an excellent opportunity to predict the path loss with the best accuracy and less time consumed by adopting ML and deep learning algorithms.

To the best of our knowledge, there is a severe gap in organizing and concluding the previously conducted efforts for path loss prediction based on ML methods. As it is well-known, all ML-based models mainly depend on the datasets for the models’ training. These datasets are brought from different environments and communication scenarios at various frequency bands for several applications. Almost all of the existing related works have proposed specific ML-based (or deep-learning-based) models and compared their performance with the traditional empirical and/or a few other ML-based path loss models to show better prediction accuracy. However, until now, there is no way to guess which ML method is the best for a specific radio propagation environment since the real measurement data will be similar in such environments. This goal can be achieved by comparing several ML-based path loss prediction models with the same datasets and input features and running away from the thinking of comparing with the traditional linear models since ML-based models already perform better according to open the literature due to the ability to create a complex non-linear relationship between their inputs and outputs. Motivated by that, this work attempts to fill this gap for a typical enclosed indoor corridor environment by providing a comparative analysis of several relevant techniques used for path loss prediction. This study will give other researchers an insight into the best ML-based model for enclosed indoor small-cell communications in the SHF and mmWave frequency regimes.

## 3. Data Preparation and Models Setup

This section provides information on the preparation of the raw data for ML-based path loss prediction. Moreover, the hyperparameters tuning method selected to achieve the best models’ performance is presented in this section. Furthermore, the justification of the models’ stability is discussed. Finally, this section presents the evaluation metrics adopted to measure and compare the models’ performance.

### 3.1. Data Preparation

The adopted real measured data were collected in a typical indoor corridor on the fifth floor of the discipline of Electrical, Electronic, and Computer Engineering, University of KwaZulu-Natal, Howard College Campus, Durban 4001, South Africa.

It is worth mentioning that we ensured that the overall measurement system was carefully calibrated, and there were no other signal transmissions in the same frequency range that were selected for this research before beginning the measurement campaigns. Moreover, to provide a high level of confidence and ensure high-quality data collection from our measurement results, the measurements were repeated and averaged for all the frequency bands and communication scenarios adopted for this paper.

As mentioned above, the wireless propagation channel selected for this research study is an indoor corridor environment that is vital and ordinarily used for many wireless indoor applications. These corridors can be analytically approximated as rectangular air-filled waveguides with dimensions immense compared to the signals’ wavelengths [10]. The dimensions of our wireless propagation channel (i.e., the indoor corridor) are 30, 1.4, and 2.63 m as the length, width, and height, respectively. The materials used to construct this corridor are bricks and dry concrete. This corridor has wooden doors to offices on one side and an elevator and a staircase on the other side [10].

The measurements were carried out for both the LOS and NLOS communication scenarios at three frequency bands from the SHF band, namely 14, 18, and 22 GHz. Furthermore, two practical heights of the transmitting antenna were selected, 1.6 and 2.3 m. The receiving antenna height was fixed at 1.6 m. The main reason for choosing the Tx antenna height to be mainly these two values (1.6 and 2.3 m above the floor level) is the fact that 1.6 m is approximately the typical user height, while 2.3 m is the height of access points in indoor environments. Moreover, these two values are the average antenna heights for these indoor environments that are adopted by many researchers [61].

The antennas used in this work are vertically polarized horn antennas with directional radiation patterns. Throughout the campaigns, the intent was to place the Tx at one end of the corridor and move the Rx away from the Tx, having a Tx–Rx separation distance of 2–24 m with an incremental step of 2 m a time [10]. In addition, the receiver was mechanically rotated in the azimuth plane to provide several angles of arrival (AoAs), which is the practical case in using the mobile as a receiver for wireless systems. More details about the measurement campaigns, channel sounder, and the environment can be found in [10].

The total number of samples collected from the measurement campaigns is 865 samples, considering all the operating frequencies (i.e., 14, 18, and 22 GHz), Tx–Rx separation distances (2–24 m with an incremental step of 2 m), antenna height values (i.e., 1.6 and 2.3 m), and AoA values (0–360 degrees with an incremental step of 10 degrees).

The raw datasets collected from the measurements were analyzed and cleaned to provide one reliable path loss value for each Tx–Rx separation distance, frequency, AoA, and Tx antenna height. Accordingly, the best features that lead to the optimum performance for the ML-based models adopted in this work are: (1) The distance between the transmitting and receiving antennas, which is the most crucial input feature that significantly affects the path loss values. (2) The operating frequency to provide multi-frequency path loss prediction models for the frequency range between 14 and 22 GHz. (3) The AoA of the Rx antenna to has LOS and NLOS characterizations of the communication’s condition. (4) The Tx antenna height that allows for more generalization of the target models.

The datasets were cleaned since this step is a vital part of any aspect in modeling based on ML algorithms. Working with impure datasets can lead to several significant challenges. On the other hand, cleaned and high-quality datasets can cause reliable models to provide outstanding results. There are many data cleaning methods, for this research, we adopted the method of removing irrelevant values and taking care of some missing values using the interpolation between the nearest two values. Hence, after cleaning and analyzing, the datasets are ready for the next step.

**Figure 1:**
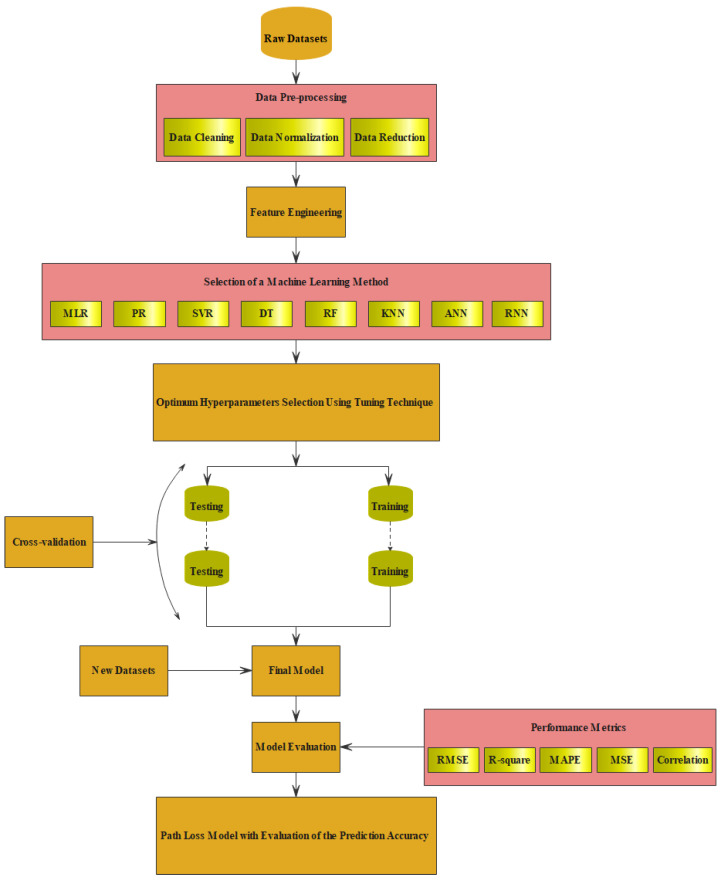
Flow chart of the adopted ML-based path loss prediction modeling technique.

After carefully selecting the input features, normalization of the data was applied for some of the ML methods. This was performed by first computing the mean value for each feature, then subtracting this mean value over the entire dataset feature to centralize the data, finally, calculating the standard deviation and dividing the subtracted values by the standard deviation. After that, the processed data were applied to each model and divided into training and testing datasets based on a reliable hyperparameter tuning technique and cross-validating the developed model to provide accurate and stable results. More specifically, of the 865 collected samples, 80% of these samples were used for training and cross-validating each model by dividing these datasets 7-fold to evaluate the model’s stability, as to be detailed in Section 3.3. The other 20% of the datasets were used for validating the models’ prediction accuracy. Figure 1 depicts the flow chart of the adopted ML-based path loss prediction strategy.

### 3.2. Hyperparameters Tuning Setup

Hyperparameter tuning techniques play an essential role in searching for the best hyperparameter for machine learning applications. Machine learning algorithms are based on complex hyperparameters that create complicated black boxes and lead to optimization challenges. Moreover, determining the hyperparameters’ values can be time-consuming if we try all possible combinations of hyperparameters. However, several techniques have been proposed to select the best hyperparameters for a particular model, including Bayesian Optimization Automate Hypermarameter Tuning (Hyperpot) [68], Spearmint Bayesian optimization [69], Sequential Model-Based Optimization (know as SMAC) [70], Autotune: A derivative-free optimization [71], Google viezier [72], Genetic Algorithm [73], and Optuna Approach [74]. These approaches aim to select the best hyperparameter that minimizes the mean square error and maximizes the accuracy, such as R-square. This is performed by training the machine learning techniques in all the hyperparameter possibilities, then selecting the ones that lead to the objective (i.e., the best performance). The tuning techniques mainly assist in looping through predefined hyperparameters and fitting the estimator (model) on the training set.

Since machine learning techniques require hyperparameter tuning to achieve both the efficiency of the model training process and the resulting model accuracy, the best hyperparameter of machine learning algorithms requires determinination. This work has applied the Optuna approach to select the best hyperparameter that leads to suitable data fitting. The Optuna approach is used in our work for multiple reasons. Optuna is a software framework for automating the optimization process of these hyperparameters. It automatically finds optimal hyperparameter values using various samplers such as grid search, random [75], Bayesian, and evolutionary algorithms [74]. It has eager search spaces that use automated searches for the optimal hyperparameter using Python conditionals, loops, and syntax. It is also a state-of-the-art algorithm that efficiently searches large spaces and prunes unpromising trials for faster results. In addition, the Optuna can parallelize hyperparameter searches over multiple threads or processes without modifying code. Finally, the best machine learning techniques depend on identifying the proper hyperparameters, avoiding both overfitting and underfitting, which is the trend of this research to achieve objective prediction accuracy.

### 3.3. ML-Based Models’ Stability

In this work, the k-folds cross-validation technique plays a significant role in assessing the machine learning model stability. The technique first divides the measured data into $k=k_{1},k_{2},\cdots ,k_{7}$ subsets, named folds. Then it trains the model on the data using $k_{1}$ to $k_{6}$ of the folds and evaluateing the model’s performance on the $k_{7}$ data. The technique continues repeating the same approach by training the model based on six-folds and evaluates the model’s performance according to the seventh (testing) fold. In each cross-validation training, the error estimation is averaged over all k trials to get the total effectiveness of the model. As can be seen, every data point gets to be in a validation set exactly once and in a training set k−1 times. This significantly reduces the bias as the model uses most of the data for fitting and reduces variance as most of the measured data are also being used in the validation set. The use of cross-validation adds a reliable stability to the effectiveness of the machine learning model since the interchange of the measured data is applied between the training and testing sets.

### 3.4. Evaluation Metrics

This subsection presents the performance metrics used to evaluate and compare the performance of the proposed ML-based path loss models. The performance metrics adopted are (1) R-squared (or $R^{2}$). (2) Root mean squared error (RMSE). (3) Mean absolute percentage error (MAPE). (4) Mean square error (MSE). (5) Correlation (Corr) coefficient. The main reason for choosing these five well-known metrics is to compare our results with the values obtained by other researchers for similar and different environments since most works in the literature adopt these metrics. The performance metrics are expressed as [7]:

(1) $$\begin{array}{c} R^{2}=1-\frac{\sum_{i=1}^{Q}{\left(PL_{i}-\hat{PL_{i}}\right)}^{2}}{\sum_{i=1}^{Q}{\left(PL_{i}-\bar{PL}\right)}^{2}}, \end{array}$$

(2) $$\begin{array}{c} \mathrm{RMSE}=\sqrt{\frac{1}{Q}\sum_{i=1}^{Q}{\left(PL_{i}-\hat{PL_{i}}\right)}^{2}}, \end{array}$$

(3) $$\begin{array}{c} \mathrm{MAPE}=\frac{1}{Q}\sum_{i=1}^{Q}\left|\frac{PL_{i}-\hat{PL_{i}}}{PL_{i}}\right|, \end{array}$$

(4) $$\begin{array}{c} \mathrm{MSE}=\frac{1}{Q}\sum_{i=1}^{Q}{\left(PL_{i}-\hat{PL_{i}}\right)}^{2}, \end{array}$$

(5) $$\begin{array}{c} \mathrm{Corr}=\frac{\sum_{i=1}^{Q}\left(PL_{i}-\bar{PL}\right)\left(\hat{PL_{i}}-\bar{\hat{PL}}\right)}{\sqrt{\sum_{i=1}^{Q}{\left(PL_{i}-\bar{PL}\right)}^{2}}\sqrt{\sum_{i=1}^{Q}{\left(\hat{PL_{i}}-\bar{\hat{PL}}\right)}^{2}}}, \end{array}$$

where *Q* is the total number of samples used for the calculation of the performance metrics, PL is the empirical path loss value, $\hat{PL}$ is the predicted path loss value, and $\bar{PL}$ and $\bar{\hat{PL}}$ are the mean values of PL and $\hat{PL}$, respectively.

## 4. Machine-Learning-Based Models

This section presents various ML-based models for predicting Path Loss for future indoor wireless communications. Eight prediction models are adopted in this work, namely MLR, PR, DT, RF, SVR, KNN, ANN, and RNN-LSTM. The following subsection will represent these models in detail.

### 4.1. Linear Regression Models

Linear Regression Models are essential techniques for addressing the regression challenges in machine learning using data modeling. The linear regression models contain various types of models and depend upon several elements [76,77,78,79]. These elements incorporate the type of target variable, the shape of the regression line, and the number of independent variables. This work adopted two types of linear regression models to predict the path loss for the selected enclosed indoor environment. These models are MLR and PR. The MLR model is a predictive model that considers more than one input to predict the target. This model identifies the correlation between the various features (dependent variables) and the target (independent variable) to find the best fit for the measured data. The MLR assumes that the inputs are $X_{1},X_{2},X_{3},\cdots ,X_{n}$, and seeks to predict the target real-value *Y*. The MLR model has the form:

(6) $$Y=f\left(X\right)=\beta_{0}+\sum_{i=1}^{n}\beta_{i}X_{i}+\epsilon ,$$

where $\beta_{i}$ are unknown coefficients (model coefficients) and ϵ defines the errors or noise. The most famous estimator that is used to estimate the coefficients model is the Least Squares (LS), in a way that it picks the coefficients $\beta_{0},\beta_{1},\beta_{3},\cdots ,\beta_{n}$ to minimize the mean squares error (MSE) presented in the following equations:

(7) $$\begin{array}{cc} \mathrm{MSE} & =\sum_{j=1}^{M}{\left(Y_{j}-\beta_{0}-f\left(X_{j}\right)\right)}^{2}, \end{array}$$

(8) $$\begin{array}{cc} \; & \;\;\;\;\;\;\;\;\;\;\;\;\;\;=\sum_{j=1}^{M}{\left(Y_{j}-\beta_{0}-\sum_{i=1}^{N}X_{ji}\beta_{j}\right)}^{2}. \end{array}$$

However, if basic expansion is made to Equation (6) by substituting $X_{2}=X_{1}^{2},$$X_{3}=X_{1}^{3}$, …, this leads to a new form of model called polynomial regression representation. A polynomial regression model can be defined as a new function that takes the form:

(9) $$Y=f\left(X\right)=\Gamma_{0}+\sum_{n=1}^{N}\Gamma_{n}X^{n},$$

where *n* is a polynomial degree of the PR model and $\Gamma_{n}$ represents the model coefficients of PR, where the LS method estimation is applied to estimate the PL model coefficient using the measured data. The polynomial regression model attempts to generate a polynomial function that estimates the measured data points. It determines the best-fit curve that passes through the entire measured data to minimize the predicted error.

The PR model is a modified version of the MLR model where the relationship between the independent and dependent variables is defined by the *n*-th degree. The best fit curve in polynomial regression passes through all the data points, depending on the power of *X* (or the value of *n*). It is recommended to analyze the turn towards the end as the higher polynomials can give undesired results.

The number of iterations adopted using the Optuna technique was 100 for both the MLR and PR methods. Moreover, the number of degrees for the PR model was 6. Finally, it is worth noting that the datasets were normalized before training and testing these models to achieve the best performance.

### 4.2. Support Vector Regression Model

Vapnike [80] proposed SVM algorithms for the binary classification problem. Later on, they worked on both multiclassification and regression problems, known as support vector classification (SVC) and SVR algorithms. The SVR applies a similar concept as the SVC algorithm with some changes. A few changes include that the target values are real numbers, the infinite possibility of which became challenging to predict using the same SVC. However, the SVR selects a boundary distance {−ϵ,ϵ} from the original hyperplane to predict the real numbers. This boundary distance is the margin of tolerance that takes only data points within this boundary. Therefore, the main goal is always similar: minimizing the prediction error and individualizing the hyperplane to maximize the margin [81].

As aforementioned, the SVR applies a similar concept to SVC, but a target variable is a real number Y∈R. As stated by Huang and Tsai [82] and Patel et al. [83], the SVR seeks the linear regression function as an alternative to finding the hyperplane in the SVC by Equation (10). This can be achieved by selecting a threshold error ϵ, which is defined to minimize the expression in Equation (11). This expression is called the ϵ-insensitivity loss error function. The SVR regression process, therefore, seeks to minimize ϵ in Equation (11) and ${\left||W|\right|}^{2}$ in the expression of *R*. The target value of the SVR method is given by:

(10) $$Y=W^{T}X+b,$$

where *Y* is the target, *W* is the coefficient, *X* is the input feature, and *b* is a constant. We define:

(11) $$|\hat{Y}{-Y|}_{\epsilon }=\left\{\begin{array}{cc} Zero, & |\hat{Y}-Y|\leq \epsilon \\ |\hat{Y}-Y|-\epsilon , & \mathrm{otherwise}\; \end{array}\right.$$

(12) $$R=\frac{1}{\epsilon }{\left||W|\right|}^{2}+C\left(\sum_{i=1}^{N}{\left|Y_{i}-{\hat{Y}}_{i}\right|}_{\epsilon }\right).$$

Tolerance variables are also introduced, defined as the value in excess of ϵ and ξ to limit the value to the regression target. Thus, the minimization of Equation (12) is updated to Equation (13), under the conditions of Equations (14) and (15) for $\xi_{i}$ and $\xi_{i}^{*}\leq 0$ and i=1,2,3,…,N. That is:

(13) $$R=\frac{1}{\epsilon }{\left||W|\right|}^{2}+C\sum_{i=1}^{N}{\left|\xi_{i}-{\hat{\xi }}_{i}^{*}\right|}_{\epsilon },$$

(14) $$\left(W^{T}X_{i}+b\right)-Y_{i}\leq \epsilon -\xi_{i},$$

(15) $$Y_{i}-\left(W^{T}X_{i}+b\right)\leq \epsilon +\xi_{i}^{*}.$$

The standard kernel functions are considered in this study, given explicitly by the linear, radial, and polynomial functions in Equations (16)–(18), respectively.

(16) $$K\left(X_{i},X_{j}\right)=X_{i}^{T}X_{j},$$

(17) $$K\left(X_{i},X_{j}\right)=e^{-\lambda ||X_{i}-X_{j}\left|\right|},\lambda >0,$$

(18) $$K\left(X_{i},X_{j}\right)={\left(X_{i}^{T}X_{j}+1\right)}^{d}.$$

**Figure 2:**
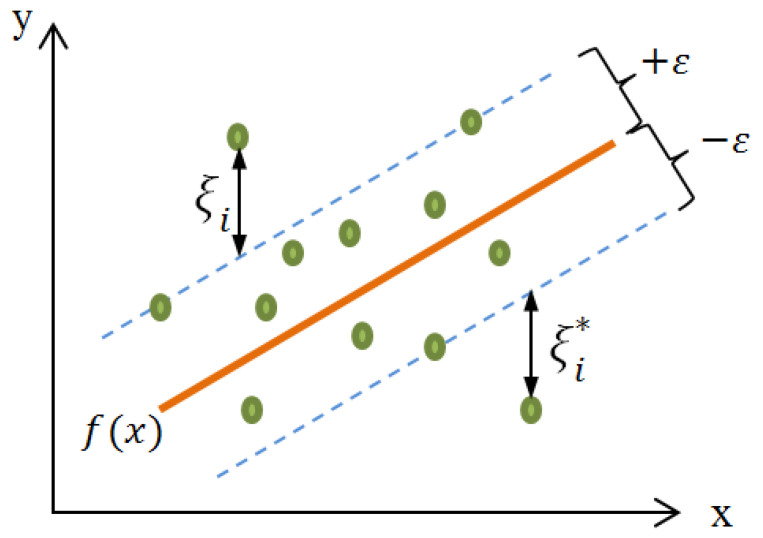
The principle of the SVR in two dimensions.

Note that the shape of the kernel function directly influences the values obtained by the SVR regression. Similarly, the constant *C* in Equation (12) and the parameters λ and *d* in Equations (17) and (18) should be optimized. For this purpose, the Optuna technique is applied to choose the optimal parameters for C,λ, and *d*, considering the lowest RMSE. According to our developed SVR model, the values of the primary hyperparameters obtained from the Optuna hyperparameter tuning technique are: (1) The kernel adopted was the radial basis function (RBF). (2) The kernel coefficient for RBF equals 0.001. (3) C=995.2783. Figure 2 shows the principle of using the SVR in two dimensions.

### 4.3. Decision Tree Regression Model

Decision tree (DT) learning plays a critical role in solving classification and regression problems. The classification and regression accuracy for its performance, when compared to existing techniques, is sufficient. The classification model learned through these techniques is represented as a tree and is called a decision tree. ID3Q [84], C4.5Q [85], and CART [86] are decision tree learning algorithms. More details can be found in [87].

The proposed decision tree creates a regression model that uses the tree structure form. It breaks down the measured dataset into small subsets while the corresponding decision tree is progressively developed, with the final output being a tree with decision nodes and leaf nodes. The decision node (an input) contains feature branches (e.g., Tx–Rx distance, AoA, Tx Height, frequency), each representing values for the attribute tested. The leaf node (e.g., the path loss value) represents a decision on the numerical target. The top-most decision node in a tree that associates with the best predictor is known as the root node. The DT regression model identifies ways to split the measured data via an algorithmic approach into smaller subsets. This approach is repeated several times until the best results are obtained. The optimum rules that lead to the best results are obtained by using variance reduction as a measure of impurity. These results are used to calculate the variance reduction for each output. A higher variance leads to a higher impurity, meaning that the corresponding conditions should be chosen as the optimum conditions for the model. Based on our model, the selected hyperparameters’ values are the MAE as a variance calculation function, and the tree has 69 nodes.

### 4.4. Random Forest Regression Model

Random forest (RF) is one of the learning algorithms that uses the tree as a base learner. The RF is introduced since a single regressor is not enough to predict the correct fit. The reason is that, based on sample data, the regressor cannot distinguish between noise and pattern, so it performs sampling with changes such that the given *n* trees to be learned are based on the dataset samples after taking the averages. The RF model sets up several trees to address the regression challenge, where each tree contains a root node, leaf nodes, and internal nodes. The root node has a set of training samples, and leaf nodes correspond to the final result. The internal nodes are split by features, and the criterion used to obtain features or split nodes is the MSE. Moreover, in the proposed setup, each tree is learned using four features selected randomly. After creating *n* trees, when the testing data is used, the decision regarding the majority of trees that come up is considered the final output. The number of trees adopted for this work in our RF model was 1090, with a maximum of 17 tree nodes.

### 4.5. K-Nearest Neighbor Regression Model

**Figure 3:**
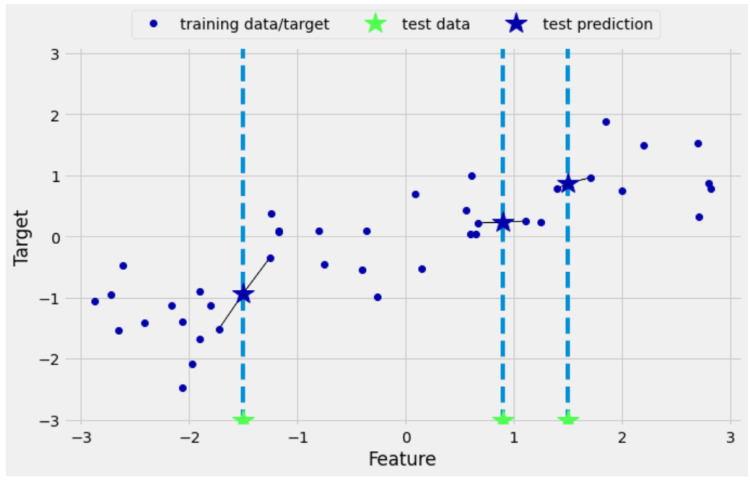
The KNN regression model with K=2.

The K-nearest neighbors algorithm is a supervised machine learning technique used to solve regression and classification problems. It is simple to understand the concept of the KNN algorithm and its application. However, it has a significant drawback of becoming significantly slow if the data size in use is increased. The KNN algorithm assumes that similar things exist in close proximity. In other words, similar things are near to each other. The KNN algorithm works by calculating the distances between a query and all the points in the data, choosing a particular number of neighbors (*K*) closest to the query, and then voting for the most frequent label (in the case of classification) or averaging the labels (in the case of regression). Selecting the correct number of neighbors (*K*) can lead to the best fit in the case of regression or classification, which can be performed by applying various *K*’s and selecting the one that gives the best results. For this hyperparameter, the best fit obtained was *K* = 2, and the distance adopted was based on Minkowski’s formula. This was achieved by using the Optuna technique using different numbers and selecting the one that best fitted the measured data. Figure 3 represents how the KNN method predicts a query using two neighbors.

The KNN calculation uses the average of the numerical target of the *K* nearest neighbors, applying one of the following distance functions:

(19) $$\mathrm{Euclidean}:\;\;\;D=\sqrt{\sum_{i=1}^{K}{\left(X_{i}-Y_{i}\right)}^{2}},$$

(20) $$\mathrm{Manhanttan}:\;\;\;D=\sum_{i=1}^{K}\left|X_{i}-Y_{i}\right|,$$

(21) $$\mathrm{Minkowski}:\;\;\;D={\left(\sqrt{\sum_{i=1}^{K}\left(\right|X_{i}-Y_{i}{\left|\right)}^{q}}\right)}^{1/q},\;q\geq 1,$$

where *X* and *Y* are the original path loss and the predicted path loss, respectively.

### 4.6. Artificial Neural Network Model

**Figure 4:**
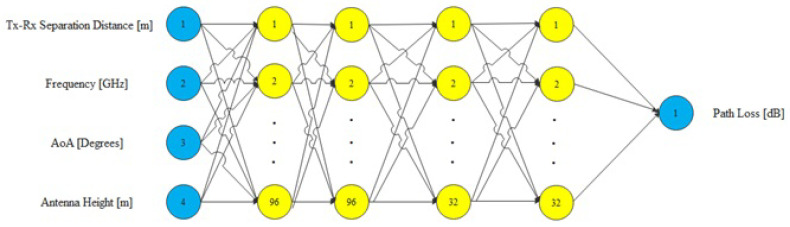
The architecture of the proposed ANN model.

Artificial neural networks have been developed based on biological neural network functionality. The ANNs are a network that contains a group of neurons, various layers, and activation functions, all of which get activated based on inputs. The proposed model is based on ANN architecture that includes four hidden layers of feed-forward neural networks. The first two hidden layers contain 96 neurons, and the last two hidden layers have 32 neurons, with each hidden layer followed by the ReLU activation function. The input for the network accepts four features from the preprocessed data. The output layer is a single neuron with a linear activation function as the transfer function that leads to the predicted value. Figure 4 depicts the architecture of our proposed ANN model. The output result is a real value representing the path loss. The hyperparameter values of the proposed ANN (number of layers, number of neurons, activation functions, learning rate = 0.001) are obtained using the Optuna technique, which gives the best hyperparameters that fit the measured data.

The proposed ANN model learns to move up or down depending on the trend feature extraction from the data, giving the fit curve. The parameter weight at every epoch is adjusted using the gradient descent with momentum to reach the global minimum error. The proposed model uses a comprehensive hyperparameter set up to identify the best weights of the parameters for the path loss prediction.

### 4.7. Recurrent Neural Network Model

The recurrent neural network based on the long-short-term memory layer is a type of ANN where the links between nodes form a directed graph along a temporal sequence. This makes it exhibit temporal dynamic behavior. Derived from feed-forward neural networks, RNNs can use their internal state (memory) to process variable-length sequences of inputs [88], which makes them applicable to tasks such as unsegmented, connected handwriting recognition or speech recognition. While the ANN output layers depend on the previous layer output to train the neural network, the RNN requires both the previous layer output and the internal state of the neural network. The internal state is defined as the output of each hidden neuron when processing the previous input observations. They are thus well-suited to process time series of data and capture their time dependencies. On the other hand, it considers the current input and the output that it has learned from the previous input for making a decision. The proposed RNN architecture is expected to extract feature representations that encode some aspects of the path loss. This new way of learning gives the RNN-LSTM model significant performance on several applications, which motivated us to select the RNN-LSTM for our work. The architecture of the proposed model is the same as the original version of RNN-LSTM; the only changes are on the hyperparameters setup.

**Figure 5:**
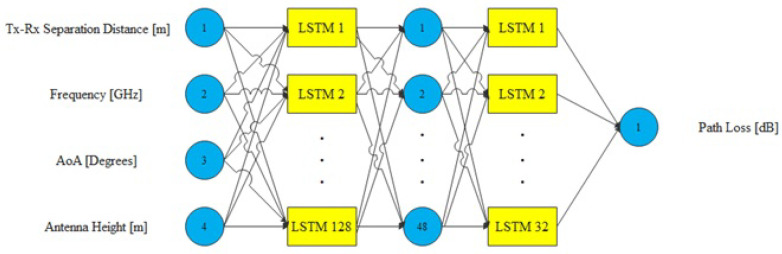
The architecture of the proposed RNN-LSTM model.

The proposed RNN-LSTM model involves the hyperparameters of two hidden layers of internal state (LSTM) and one feed-forward neural network layer. The first and last LSTM hidden layers have 128 and 32 neurons, respectively. The feed-forward neural network layer contains 48 neurons. Each hidden layer is followed by the ReLU activation function. The model uses a learning rate of 0.0001 to train the model. The input for the network accepts four features from the preprocessed data. The output layer is a single neuron with a linear activation function as the transfer function. The output result is a real value representing the path loss. The cost function is used to optimize the mean square error. Figure 5 shows the architecture of the proposed RNN-LSTM model. It should be noted that the RNN-LSTM model is more complex compared to the ANN model and has more parameters to train [89,90].

## 5. Results and Discussions

**Figure 6:**
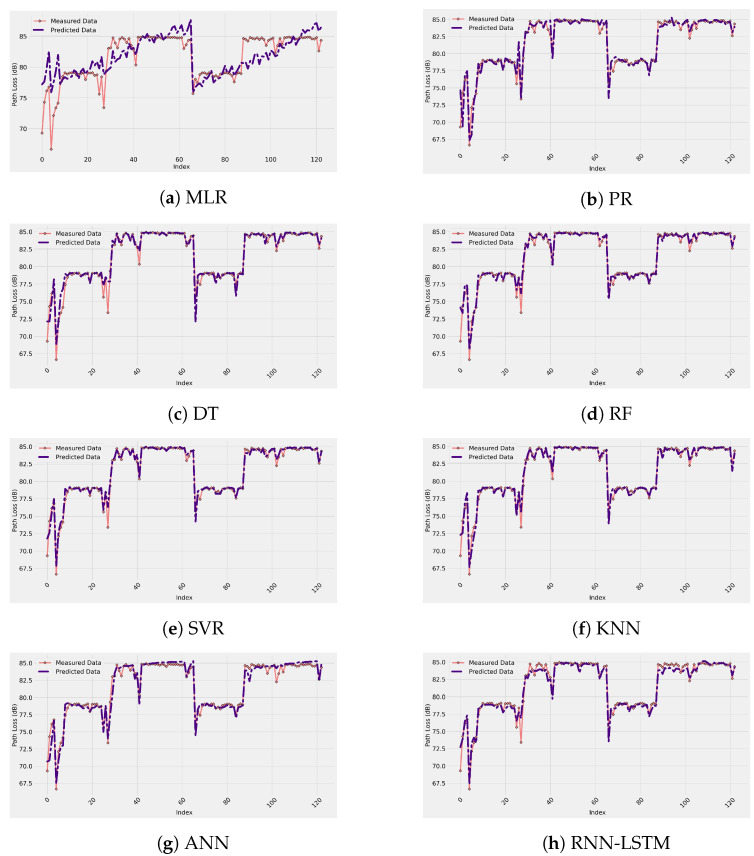
Measured and predicted path loss data for each ML-based model.

Figure 6 depicts the measured data and ML-based path loss prediction models for the enclosed indoor wireless channel selected in this work. These results are for 20% of the measurement data used for testing the performance of the models. As described above, the input features of all the ML-based models adopted for this study are the Tx–Rx distance, operating frequency, AoA, and the Tx antenna height. From Figure 6, all the models (except the MLR) fit the real measurement data accurately. Furthermore, it is clear from the figure that there is a significant match between the measurement data and the predicted models, which means a high prediction accuracy was provided by these models. Based on numerical analysis, the R-squared values of all the models fall between 0.4704 and 0.9798, while the RMSE values are in the range of 0.0216 to 2.9008 dB.

Moreover, the MAPE values are between 0.37% and 6.94%, and for the correlation factor, the values are in the range of 69.45% to 99.07%. Thus, all the previous metrics results show the quality of the model’s predictions for such environments. The reasons behind the accuracy of these models are: (1) The availability of training data since 80% of the measurement data was used to train the models. (2) Efficient input feature selection that considers crucial factors, such as the AoA and the Tx antenna’s height for these indoor environments, in addition to essential factors, such as the Tx–Rx separation distance and the multi-frequency operating range of 14 to 22 GHz. (3) The use of the hyperparameter tuning technique, namely Optuna, to choose the best values of the hyperparameters (for example, type of activation function, number of layers, number of neurons, learning rate, number of trees) instead of choosing them manually, which leads to a minimum prediction error. (4) The preprocessing of the data for some models, such as the ANN and RNN-LSTM.

As a comparison between the models, the ANN model provides the best average RMSE value, while the worst is for the MLR model. However, the RNN-LSTM, KNN, RF, DT, SVR, and PR show their ability to predict path loss since their average RMSE values were less than 1.1653 dB. This can be validated from the R-squared and the correlation coefficient values that show a minimum of 0.8690 and 0.9342, respectively, close to the ideal value of 1. Table 1 provides the performance metric values of the selected ML-based models. It is worth noting that the table provides three values of each metric, the minimum, average, and maximum value. These values came from the cross-validation technique adopted for this study that divided the measurement data into seven folds to evaluate the stability of each model. It can be seen from the table that the models provide highly stable results since the deviation of each metric from its average to maximum or minimum is small. Furthermore, the results displayed in Table 1 are based on all values of the frequency bands selected for this research (i.e., our ML path loss models are multi-frequency), which means that these models have the ability to accurately predict the propagation loss at the adopted frequency regime.

**Figure 7:**
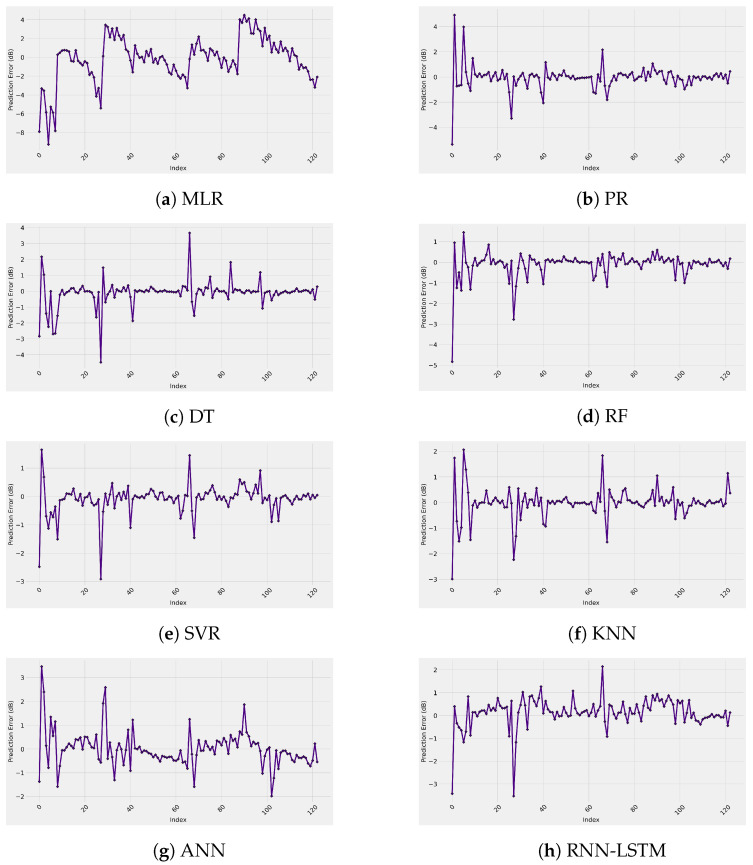
Prediction error of each ML-based model.

**Figure 8:**
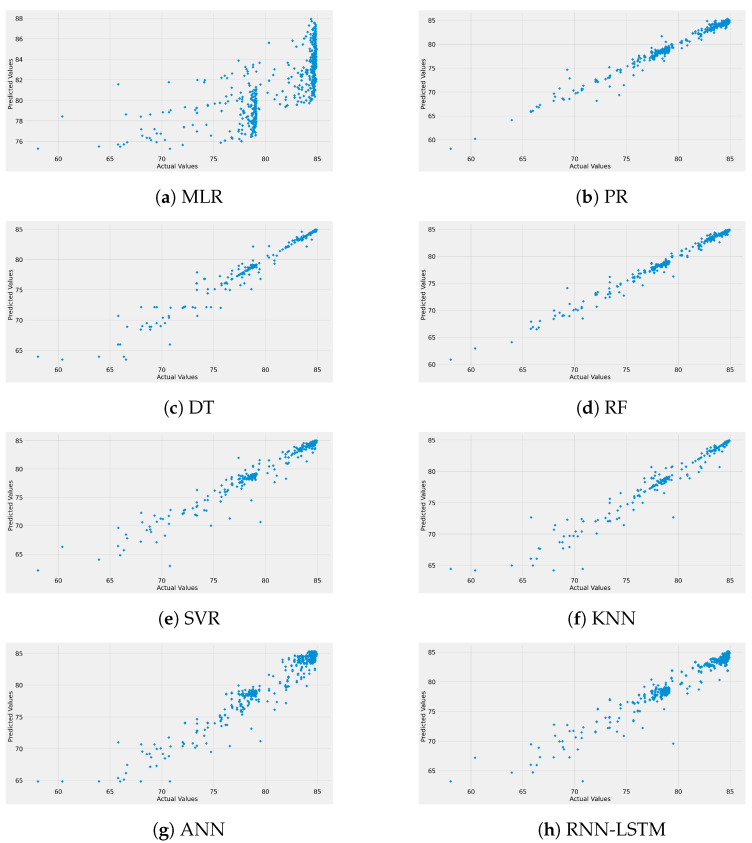
Measured vs. predicted path loss data for each model.

Figure 7 represents the test set prediction error of the ML-based models used in this work. In addition, the figure provides insights into the range of the difference between the measured and ML-predicted path loss models. These curves observe random distributions with average error values around zero with error impulses of 6 dB, as shown in the worst case of the MLR model. This means that the predicted ML-based path loss value has a maximum of only 6 dB difference from the real measured path loss value. Finally, the predicted and actual path loss values are given in Figure 8. Again, the figure proves what we have discussed in this section: all the models have a significant prediction performance with the best accuracy provided by all the models since there are clear straight lines, except the MLR model with an inaccurate performance.

**Figure 9:**
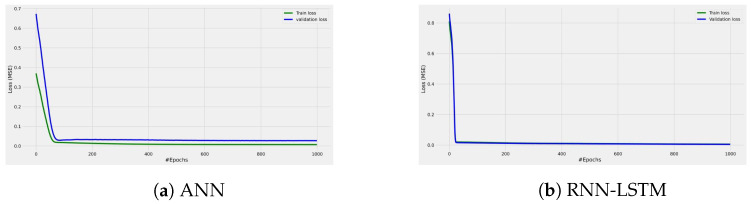
Training and validation loss for both ANN and RNN models.

**Figure 10:**
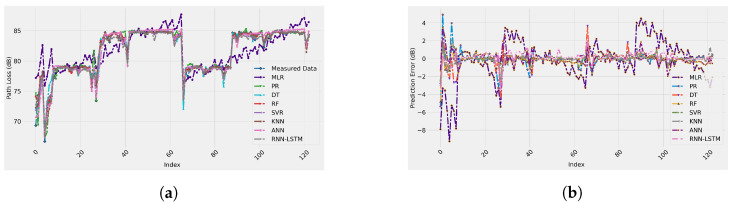
Measured and predicted path loss and the prediction error of all the ML-based models. (**a**) Measured and predicted path loss; (**b**) Prediction error.

The validation and training loss in Figure 9 shows the excellent fitting of the ANN and RNN models since the validation curve is slightly higher than the testing curve in the case of the ANN model, while the validation curve matches the testing curve across all the epoch values in the case of the RNN-LMTS model. The results reveal that the structure of these neural networks provides high precision in fitting the measurement data without underfitting or overfitting issues. Figure 10 depicts the measured and predicted path loss and the prediction error of all the ML-based models selected in this work.

Runtime analyses of ML models are essential for understanding the complexity of machine learning algorithms. It is crucial for algorithm selection in specific tasks and vital for successful implementation. Therefore, it is always a good practice to do runtime analysis and comprehend the complexity of ML algorithms. Runtime analysis can be seen from two directions: time complexity and space complexity. Time complexity measures how fast or slow a model performs the task, while space complexity is the amount of memory required to execute the task. In this work, time complexity analysis is performed to study the comparison between the adopted ML models. Table 2 represents the runtime of each model where some models take less than a second to complete the task, such as MLR, PL, DT, and KNN, while others take more time to finish the task. More specifically, as depicted in Table 2, the minimum runtime was achieved by the MLR model, 21.3 ms, which means that the MLR model has the least complexity among the other models. Nevertheless, the MLR model has the worst performance according to the results presented in Table 1. The highest runtime was observed from the run of the RNN-LSTM model since it took almost 125 s to train the model. In general, the runtime obtained by our ML models is comparatively short, which indicates that the adopted models can fit the path loss problems with relatively low complexity. However, the runtime varies according to the performance of the computer used.

The experimental platform is on a PC with an Intel Core i7 processor, Gen (10) 1.20 GHz cores, 64-bit operating system, and an x64-based processor. It also has 1 TB shared memory and 16.0 GB RAM. The software used for the model implementation includes Python Version 3.5.2, Tensor flow backend 1.1.0, and TFlearn 0.3. The adopted algorithms have been used from two build-in python libraries called Scikit-learn (Sklearn) and Tensorflow (TF). These are the most useful and robust libraries for machine learning in Python. It provides efficient tools for machine learning modeling, such as classification, regression, and clustering. Training and testing times for the results are provided in Table 2 for each model.

To investigate the impact of choosing the antenna height as an input feature, we removed the Tx antenna height column from the datasets’ input features. Table 3 provides the performance metrics’ values when the adopted ML-based models have three input features: the Tx–Rx separation distance, operating frequency, and the AoA. It is clear from the results that the overall performance of the models became worse than when the models had all four input features. For example, the average R-squared and the correlation coefficient values are reduced by approximately 3% and 4%, respectively, while the average RMSE value was increased by 1.6942 dB. For the RNN-LSTM model, the average RMSE was also increased by 1.3731 dB. The results presented in Table 3 indicate the importance of considering the Tx antenna as an input feature.

Furthermore, the impact of only having the separation distance and frequency as the two input features (removing the Tx height and the AoA) is investigated. Table 4 shows the values of the performance metrics after removing both the Tx height and the AoA from the input features. Again, the accuracy of the models became worse compared to the results provided in Table 1 and Table 3. For instance, the average R-squared value and the correlation coefficient reductions are approximately 9.3% and 5.2%, respectively. In addition, the average RMSE value was increased by 1.9673 dB for the ANN model, while for the RNN-LSTM model, the matter was increased by 1.9808 dB. Again, the results reveal the effectiveness of having the four input features together.

Based on the results provided, it is clear that the careful selection of the input features for training the models can ensure high prediction accuracy without considering more features, which leads to less complexity in using the proposed models for such environments. However, there is a high demand for developing precise mechanisms capable of generalizing the results for data far different (representing other communication environments) from the data used for training the models, to overcome the need to conduct extensive measurement campaigns in all the possible communication scenarios. Furthermore, these generalizing mechanisms will allow for adaptive data-driven prediction models that accurately represent the channel characteristics for future mobile networks relying on fewer training samples, which leads to faster and more cost-effective planning of the wireless systems. As a suggestion for expanding the datasets, our improved CI and FI models [10] can be used in this manner since they provide higher precision than the standard models in terms of fitting the real measurement data for path loss prediction.

Future research will be directed toward developing a ML-based path loss model that provides better accuracy and stability than other well-known methods. Using the results from this study that the best performance was achieved by the neural networks (i.e., ANN and RNN-LSTM), prediction based on deep neural networks such as using a convolution neural network (CNN) will be investigated. Finally, the impact of the LOS probability will also be considered to provide probabilistic path loss models based on neural networks.

## 6. Conclusions

Due to the characteristics of the wireless signals at the SHF and mmWave frequency bands compared with today’s sub-6 GHz frequency regime, providing accurate and stable path loss prediction models is a challenging problem. Motivated by that, this work considered an extensive comparative analysis to evaluate the performance of the most widespread and used machine learning methods, namely the MLR, PR, RF, DT, SVR, KNN, ANN, and RNN-LSTM. The data adopted for this research were collected in a typical indoor corridor environment at 14, 18, and 22 GHz frequency bands. The input features used to train the models were carefully selected: the Tx–Rx separation distance, frequency, Tx height, and the AoA. To ensure reliable and stable results, we used a cross-validation technique to divide the data into seven folds of training and testing datasets and provide the minimum, average, and maximum results. Moreover, we utilized a hyperparameters tuning method to select the optimum hyperparameters of the model and avoid the time consumption of the manual selection. Furthermore, five performance metrics were applied to evaluate the models: R-squared, RMSE, MAPE, MSE, and the correlations coefficient. The main results obtained from this work reveal that all the adopted models (except the MLR) have accurate and stable performances in predicting the path loss for enclosed indoor environments, such as corridors. Moreover, as the comparison between the models, the best-fit models according to the minimum RMSE and high R-squared and correlation factor are the ANN and RNN-LSTM. Finally, this work shows that these ML-based models could be promising solutions with higher precision for predicting path loss for future indoor wireless communication networks. The future scope of this work will be to evaluate deep learning methods and provide generalization techniques that will help estimate the path loss in complex environments without the need to conduct massive measurement campaigns.

## Figures and Tables

**Table 1:** Performance metrics’ values of all the ML-based models selected.

Models	$R^{2}$			RMSE			MAPE			MSE			Corr		
	**Min**	**Avg**	**Max**	**Min**	**Avg**	**Max**	**Min**	**Avg**	**Max**	**Min**	**Avg**	**Max**	**Min**	**Avg**	**Max**
MLR	0.4704	0.5220	0.5711	2.5191	2.7713	2.9008	0.0220	0.0239	0.0252	6.3461	7.6943	8.4144	0.6945	0.7274	0.7573
PMR	0.8743	0.9286	0.9556	0.8721	1.0480	1.2856	0.0059	0.0072	0.0087	0.7605	1.1248	1.6528	0.9550	0.9679	0.9781
SVR	0.9054	0.9414	0.9798	0.5369	0.9252	1.1586	0.0037	0.0054	0.0064	0.2882	0.8952	1.3423	0.9520	0.9731	0.9907
DT	0.8690	0.9125	0.9473	0.8678	1.1653	1.5263	0.0052	0.0068	0.0085	0.7531	1.3988	2.3296	0.9342	0.9606	0.9743
RF	0.9189	0.9461	0.9688	0.6680	0.9145	1.2534	0.0041	0.0056	0.0070	0.4463	0.8688	1.5709	0.9669	0.9761	0.9853
KNN	0.9068	0.9443	0.9756	0.5909	0.9175	1.2163	0.0040	0.0052	0.0058	0.3492	0.8787	1.4794	0.9524	0.9747	0.9877
ANN	0.9017	0.9352	0.9755	0.0270	0.0355	0.0435	0.0220	0.0312	0.0587	0.0007	0.0013	0.0019	0.9530	0.9732	0.9878
RNN-LSTM	0.8889	0.9160	0.9762	0.0216	0.0418	0.0592	0.0161	0.0390	0.0694	0.0005	0.0019	0.0035	0.9433	0.9666	0.9882

**Table 2:** Runtime comparison of the adopted ML models.

Model	RunTime (Seconds)
MLR	0.0213
PR	0.5251
SVR	3.8647
DT	0.1379
RF	11.2477
KNN	0.0782
ANN	59.1485
RNN-LSTM	124.9184

**Table 3:** Performance metrics after removing the antenna height from the input features of the models.

Models	$R^{2}$			RMSE			MAPE			MSE			Corr		
	**Min**	**Avg**	**Max**	**Min**	**Avg**	**Max**	**Min**	**Avg**	**Max**	**Min**	**Avg**	**Max**	**Min**	**Avg**	**Max**
MLR	0.4072	0.5227	0.5903	4.4745	5.0024	5.2940	0.5178	0.5409	0.6281	20.0212	25.024	28.0272	0.6091	0.6998	0.7703
PMR	0.6479	0.7324	0.8261	3.2093	3.3903	3.8639	0.4212	0.324	0.5472	10.2993	11.4943	14.9299	0.6503	0.7477	0.8451
SVR	0.8022	0.8432	0.8792	2.3015	2.6190	2.7020	0.0936	0.0753	0.1063	5.2969	6.8592	7.3010	0.8502	0.8734	0.8904
DT	0.7882	0.8161	0.8577	2.1743	2.2942	2.6169	0.0851	0.1066	0.1380	4.7276	5.2635	6.8480	0.8300	0.8606	0.8789
RF	0.8837	0.9048	0.9384	1.4678	1.9786	2.2862	0.0825	0.0946	0.1064	2.1545	3.9147	5.2266	0.8785	0.9028	0.9451
KNN	0.8636	0.9111	0.9532	1.5418	1.7558	2.2647	0.0742	0.0857	0.0969	2.3772	3.0829	5.1288	0.9048	0.9281	0.9567
ANN	0.8528	0.9093	0.9374	1.4144	1.7297	1.9756	0.0797	0.0801	0.0894	2.0006	2.9918	3.9030	0.9187	0.9382	0.9685
LSTM	0.7537	0.8509	0.8971	1.4146	1.4149	1.4160	0.0793	0.0893	0.0969	2.0011	2.0021	2.0051	0.8845	0.9062	0.9441

**Table 4:** Performance metrics after removing the antenna height and the AoA from the input features of the models.

Models	$R^{2}$			RMSE			MAPE			MSE			Corr		
	**Min**	**Avg**	**Max**	**Min**	**Avg**	**Max**	**Min**	**Avg**	**Max**	**Min**	**Avg**	**Max**	**Min**	**Avg**	**Max**
MLR	0.3580	0.4367	0.5379	4.9492	5.4129	5.9942	0.6272	0.7240	0.8212	24.4943	29.2993	35.9299	0.4983	0.5658	0.6692
PMR	0.5707	0.6344	0.7385	3.3198	4.0033	4.0034	0.5978	0.6429	0.7181	11.0212	13.024	16.0272	0.6098	0.6343	0.7044
SVR	0.7562	0.7732	0.7943	2.9204	3.2991	3.5713	0.1097	0.1443	0.1720	8.5288	10.8839	12.7539	0.7970	0.8264	0.8650
DT	0.6648	0.7298	0.7696	2.5871	2.9862	3.2512	0.1198	0.1443	0.1697	6.6935	8.9175	10.5706	0.6633	0.7250	0.8088
RF	0.8211	0.8512	0.9092	1.8355	2.1076	2.6163	0.1064	0.1101	0.1345	3.3690	4.4421	6.8452	0.8447	0.8780	0.9026
KNN	0.7937	0.8455	0.8832	2.1794	2.2420	2.8016	0.1009	0.1272	0.1324	4.7498	5.0264	7.8486	0.8419	0.8814	0.9150
ANN	0.8089	0.8482	0.9052	1.9763	2.0028	2.4527	0.0988	0.1049	0.1364	3.9056	4.0114	6.0158	0.8973	0.9224	0.9306
LSTM	0.7456	0.8240	0.8976	1.7334	2.0226	2.4280	0.0839	0.0998	0.1291	3.0049	4.0910	5.8954	0.8686	0.9278	0.9499

## Data Availability

The data that support the findings of this study are available from the corresponding author, (M.K.E.), upon reasonable request.
